# The Influence of Urban Gardening Activities on Participants’ Perceived Restorativeness, Resilience, Sense of Community and Stress

**DOI:** 10.3390/healthcare11121664

**Published:** 2023-06-06

**Authors:** Hee-Ran Kim, Won-Suk Oh, Jin-Gun Kim, Won-Sop Shin

**Affiliations:** 1 Graduated Department of Forest Therapy, Chungbuk National University, Cheongju 28644, Republic of Korea; veryrosemoss@daum.net; 2 Agricultural Technology Center of Hwaseong City, Hwaseong 18583, Republic of Korea; zirisarang@naver.com; 3 Korea Forest Therapy Forum Incorporated Association, Cheongju 28644, Republic of Korea; k64804171@gmail.com; 4 Department of Forest Sciences, Chungbuk National University, Cheongju 28644, Republic of Korea

**Keywords:** urban garden, perceived restorativeness, resilience, sense of community, stress, salivary cortisol

## Abstract

This study aimed to investigate the influence of urban garden activities on participants’ perceived restorativeness, resilience, sense of community, and stress reduction. A total of ninety individuals who agreed to participate in the experiment were divided into experimental and control groups. To collect data, 16 sessions of urban garden activities were conducted every two weeks from May to November 2022. Perceived Restorativeness Scale, Connor–Davidson Resilience Scale, Sense of Community Index, and Brief Encounter Psychosocial Instrument were employed to measure participants’ psychological effects. To evaluate physiological effects, salivary cortisol tests were performed. The results of the study revealed that urban gardening activities influenced on participants’ physiological and psychological reactions in positive ways.

## 1. Introduction

According to the “2019 Urban Planning Status Statistical Survey”, 9 out of 10 Koreans reside in urban areas. The proportion of the urban population increased from 50.1% in 1970 to 81.9% in 1990 and reached 91.8% as of 2019 [1]. Urbanites suffer from various stresses due to environmental stress sources. To combat this problem, various types of gardens have been created in cities [2].

According to the Garden Act enacted in 2015, the social demand for gardens is expanding nationwide in Korea. Therefore, the act prescribes and supports garden industrialization and establishing a basic plan for fostering and expanding gardens [3]. As interest in nature and well-being culture spreads and nature-friendly leisure activities increase, movements are being made to encourage residents to actively meet citizens’ educational needs for plants and gardens. In order to systematically support the gardening activities, the Korea Forest Service established a legal and policy foundation [4].

Urban gardening refers to planting various types of plants in a city or preparing an environment related to them and comprehensively refers to gardens created in the area of a city [5]. Urban gardens can be understood by including examples of urban agriculture, container gardens, community gardens, indoor gardens, guerrilla gardens, and rooftop gardens [6]. The gardens in modern cities are places where ‘participation’ through ‘priming behavior’ is emphasized. City’s garden value is highlighted as a ‘place of productive function’ to grow and eat crops, an ‘alternative green’ to reduce food mileage, and a ‘place to restore community life’ [7]. It also emphasizes that an urban garden should highlight community and cultural values. Therefore, public participation in planning, managing and garden activity is important [8].

Alison et al. [9] explained the importance of urban gardens to maintain and improve urban biodiversity, benefit human health and welfare, and connect people to the natural world. Park and Jeong [10] explain the effect of urban gardens, and citizens close to the gardens contribute to their ability to restoration from natural factors such as grass, flowers, plants, and water, which not only contribute to psychological well-being, but also low stress, and increased exchanges with neighbors. The garden positively affects urban aesthetics and food and desert issues, such as safety, cleanliness, and greening of alleys, and has a significant psychological and social effects. The garden is said to be an efficient means of improving mental and physical health because it is accessible to urban residents and provides as many psychological benefits as forests.

Humans have social relationships from birth and live in the community through participation and cooperation to solve the increasingly complex problems of modern society. Mcmillan and Chavis [11] defined community consciousness as a sense of belonging, feeling the problem of members as an individual or group/social problem. Among many studies, Kim [12] argued that an urban garden could be a community or social place which could revitalize the symbol of community. Sim and Zoh [7] also reported that an urban garden is a place to relieve daily stress from urban society. They suggested to secure small portions of areas in Seoul to develop urban Ssamju (small size) garden.

In modern society, people are involved in many stressful works of daily life, and easily feel tired and pressured physically and mentally [13]. Therefore, individuals engage in restorative activities and experiences such as visitation to natural environments or staying in natural areas [14]. According to the Attention Restoration Theory (ART) proposed by Kaplan and Kaplan [13], suggests the ability to concentrate may be restored by exposure to the natural environment. Research on the restorative environment has focused on natural and forest environments located far from urban areas. In a study by Shin et al. [15], the natural environment positively affected psychological restoration. They also argued a positive correlation between the preference and psychological restoration of the visiting areas. However, research on the restorative environment based on gardens in urban areas is still insufficient.

Resilience is a fundamental coping function that can restore psychology against an individual’s adversity [16]. Lazarus and Falkman [17] stated that people’s psychological well-being is threatened by stress beyond their ability to cope with. Several previous studies emphasized the relationship between stress levels and resilience. Lee et al. [18] suggested that healing agricultural activity positively affect stress relief and resilience in young adults. Kim [19] also reported that leisure satisfaction in urban parks positively affects job stress and resilience of office workers.

Ulrich [20] suggested that simply looking at nature is more effective in promoting stress reduction than looking at urban environments devoid of trees and green nature. Honeyman [21] argues urban environments with grass and trees are significantly more restorative than urban scenes without nature. In 2021, about 29% of adults aged 19 or older in Korea expressed they were heavily stressed, and 10–13% of them experienced depression over the past ten years [22]. It can be seen that stress and depression are continuously occurring in modern daily life. As one of the solutions to combat increasing social diseases, the importance and necessity of healing garden activities and programs with community-based public is increasing. Park et al. [23] emphasize the function and effectiveness of garden healing as a critical strategy for green welfare and healing as public interest and social awareness of garden activities increase.

Garden activity, as a nature-friendly intervention therapy for public psychological and emotional well-being, is emerging among various areas in Korea. Despite the current interest in implying garden activity as a public health promotion, research on the value and meaning of urban gardens is insufficient. Therefore, this study aims to add evidence of psychological effectiveness from participating in garden activity.

## 2. Materials and Methods

### 2.1. Study Site

**Figure 1:**
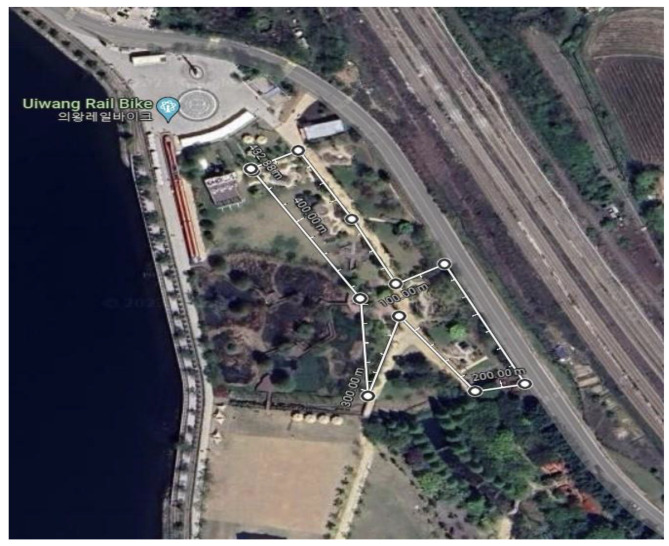
Rainbow Light Garden (The site of the experiment indicated by line; https://www.google.co.kr/map (accessed on 20 May 2023)).

To collect data, series of experiments (the urban garden activity program) were conducted at the Rainbow Light Garden located in Hyangnam-eup, Hwaseong-si, Gyeonggi-do in Korea (Figure 1). The rainbow garden is located between apartments, shopping malls, and new sites in agricultural cities created by developing farmland and hills and is located on a circular concrete floor with a diameter of 80 m and an area of 2355 m^2^. The main species are Lonicera caerulea, Cornus kousa, Hydrangea macrophylla, Cotinus coggygria, Akebia quinate, Rosa pendulina, Kerria japonica, Clematis patens, and there are box gardens planted with shrubs, vineyards, bulbs and seasonal flowers, and vegetable gardens for donation and sharing to the socially disadvantaged.

### 2.2. Participants

The sample size of this study was determined by substituting the effect size, significance probability, and power using the G-Power 3.1.9.7 Program (University of Düsseldorf, Düsseldorf, Germany). The effective size and dropout rate of 20% were referred to Lee [24], a previous study, and the sample size was 78 when the F-test calculated as Effect size 0.23, alpha value 0.05, and 1-β value Cohen’s power was 0.95. In this study, the sample size was set to 90 participants in consideration of the dropout rate.

This study investigated the value of the garden as a psychological restorative environment through urban garden activities. To investigate the psychological effectiveness of the garden activity, the participants were randomly divided into three groups, experimental group 1, experimental group 2, and a control group. The data collection period spanned from 1 April to 20 April 2022, with garden activity participants at Hwaseong Agricultural Technology Center, Gyeonggi-do. This study was conducted with the deliberation and approval of the Institutional Review Board of Chungbuk National University (IRB Number: CBNU-202203-HRBRHR-0045).

Participants were recruited by posting notices throughout community centers, and notices in the local newspapers. The inclusion criteria for recruiting the participants who were eligible for the study were: (1) no diagnosis of reaction to severe stress and/or a depressive episode; and (2) could not be suffering from any drug or alcohol abuse. In addition, KRW 30,000 (USD 30.00) was offered to every participant who faithfully participated in and complied with all experiment schedules.

A total of 94 volunteers were recruited to participate in the experiment. However, four volunteers failed to complete all experiments scheduled, so data from 90 participants were analyzed for this study.

We employed the “pre-test–post-test control group design” and used a control because it is practically impossible to eliminate all bias and outside influence that could alter the results of the experiment. To secure homogeneity between the experiment and control groups, the participants were randomly distributed into each group.

Experimental groups 1 and 2 conducted 16 garden activity programs once every two weeks from May to November 2022. The control group consisted of 30 participants who did not participate in the garden activity program during the same period to secure the homogeneity of the group. The garden activity program conducted in this study was planned and carried out by two gardeners and researchers with qualifications as landscape technicians. The urban garden activity program was divided into experimental group 1 and experimental group 2. Participants in the control group maintained their normal life.

This study investigated the effect on participants’ perceived restorativeness, resilience, sense of community, and stress through the garden activity program 1 (planting, caring for, and managing plants), and the garden activity program 2 (program 1 + forest healing therapy). The experimental design was carried out using the pre-test–post-test control group design method of experimental group 1, experimental group 2, and the control group. Program 1 was conducted for experimental group 1, and the effect was investigated. The treatment effect was evaluated by pre–post-testing with Perceived Restrictiveness Scale, Connor–Davidson Resilience Scale, Sense of Community Index, Brief Encounter Psychosocial Instrument, and saliva cortisol. Table 1 shows a schematic diagram of the research design for program effectiveness verification.

### 2.3. Psychological and Physiological Assessment

This study conducted with a self-reported survey on demographic questions, the Perceived Restorativeness Scale (PRS), the Connor–Davidson Resilience Scale (CD-RISC), the Sense of Community Index (SCI), and Brief Encounter Psychosocial Instrument (BEPSI). Measurement of saliva cortisol, a stress hormone, was performed as a physiological indicator of stress level.

The Korean version of the Perceived Restorativeness Scale developed by Hartig et al. [25] and adapted by Lee and Hyun [26], which measures how much a specific environment is equipped as a restorative environment depending on the subject was employed for measuring psychological effectiveness. This scale consisted of four sub-factors: rest, fascination, organization, and understanding. The scale has a total of 26 questions, and each question has Likert 7-point answer category from “not at all (1 point)” to “very much (7 points). The reliability of this study was Cronbach’s α = 0.94.

The Korean version of the Connor–Davidson Resilience Scale (CD-RISC), developed by Connor and Davidson [27], was employed to measure participants’ resilience. The resilience scale used previous studies such as Ahn’s investigation for the elderly’s well-being [28]. This measure consists of sub-dimensions of resilience: inner strength, patience, optimism, a capacity for change, a sense of control over the environment, and spirituality, a belief in spiritual influence. The scale comprised a total of 25 questions, from “not at all (0 points)” to “almost always (4 points)”. The reliability of this study was Cronbach’s α = 0.90.

The Sense of Community Index (SCI) developed by McMillan and Chavis [11] was used to measure the participant’s sense of community. This scale comprises four sub-factors: satisfaction of needs, member consciousness, mutual influence consciousness, and emotional intimacy. With a total of 12 questions with 5-point Likert category. The reliability of this study was Cronbach’s α = 0.75.

Participants’ stress levels were measured by the Brief Encounter Psychosocial Instrument (BEPSI) developed by Frank and Zyzanski [29]. The Korean version of the scale was modified by Bae et al. [30] and Yim et al. [31]. This scale measures the degree of stress feeling state of the participants’ with five questions on a Likert 5-point scale. The reliability of this study was Cronbach’s α = 0.82.

As a physiological indicator of stress, saliva cortisol levels were measured twice before and after the study, and in the middle of the study. In the middle of the study, two measurements were conducted to reduce the variables of the study and increase reliability. Cortisol varies with a 24-h cycle, so saliva was collected at the same time between 9:30 a.m. and 12:00 p.m. when the program started and ended. Since saliva cortisol is easily contaminated by food and gum bleeding, food intake and brushing were restricted from an hour before saliva collection. For saliva collection, referring to the study of Kim [32], a highly accurate and widely used polyester or polypropylene Salivette System was used. About 2 mL of saliva samples were collected by putting an absorption swab under the tongue of the participant, and the researcher delivered the samples in a frozen state to the research institute. In the laboratory, experts centrifuged the saliva absorbed by the swab of the saliva container. They then analyzed it on ELISA Reader (BioTek, Winooski, VT, USA) devices using Human Cortisol ELISA Kit (DRG, Springfield, NJ, USA) reagents.

### 2.4. Program

**Figure 2:**
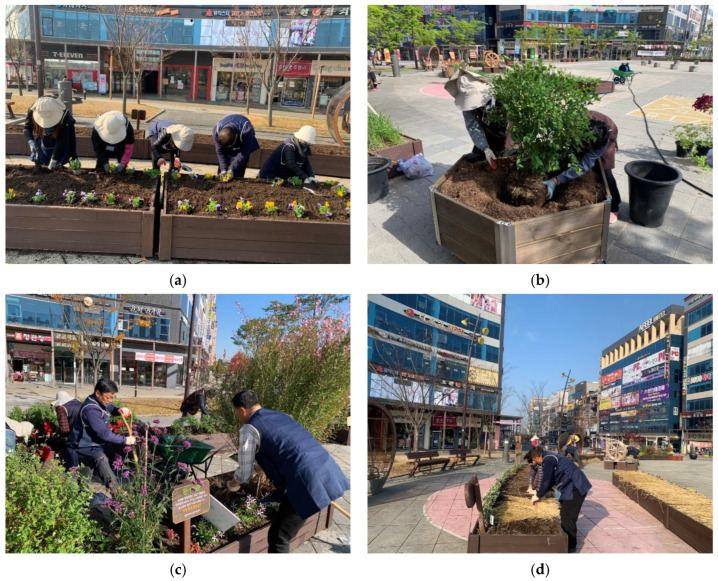
Garden activities program I. (**a**) Planting spring flower; (**b**) Placement and Planting of Trees; (**c**) Garden care; (**d**) Garden Plant Overwintering care.

**Figure 3:**
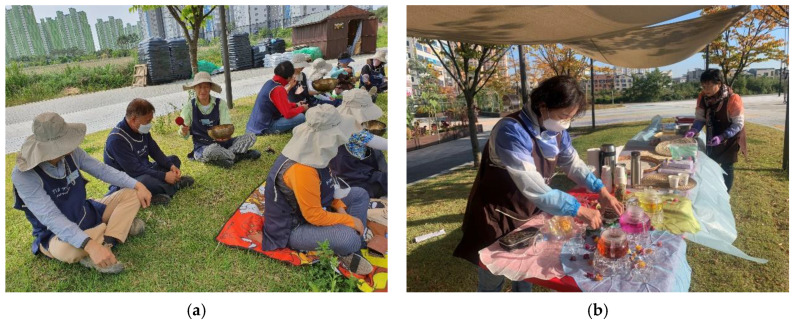

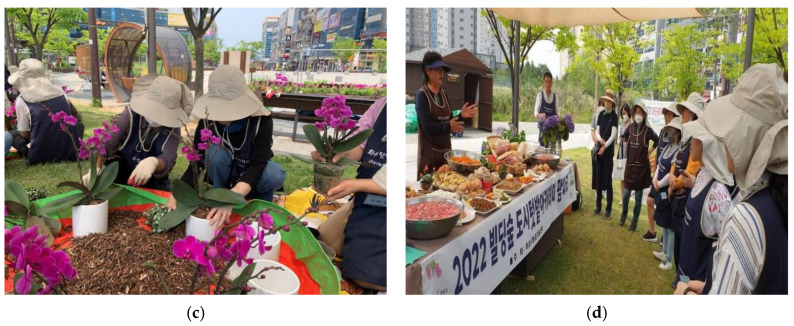
Garden activities program II. (**a**) Singing Bowl meditation; (**b**) Flower tea therapy; (**c**) Phalaenopsis flowerpot making; (**d**) Healthy Meal Garden Farm Party.

The urban garden activity program consisted of 16 sessions to increase awareness of the restorative environment, resilience, community consciousness, and reducing stress. The garden activity program was composed of planting plants, trees and shrubs, vineyards, vegetables, fruits, flowers, and bulbous plants directly, managing flowers in various seasons, and inducing interest in the garden (Table 2). Experimental group 1 conducted a garden activity program I (Figure 2), and experimental group 2 performed a garden activity program II (Figure 3) that added five senses stimulation activities such as plant therapy for growing plants, diet for garden products, aerobic exercise and body relaxation, and meditation to help emotional stability. The program was operated by one forest healing instructor, one main instructor qualified as a landscaping engineer and three urban agricultural managers. The specific program schedule is shown in Table 2.

### 2.5. Data Analysis

This study was conducted with experimental and control groups to determine the effect of urban garden activity programs on participants’ perception of the restorative environment, resilience, community consciousness, and stress. For data analysis, 90 questionnaires were analyzed using the SPSS 19.0 statistical program. Frequency analysis and multiple response analysis were conducted for the demographic characteristics and the degree of demand for the number of study subjects. Repeated measures analysis of variance (RM ANOVA) was conducted to verify the means difference between the experimental group and the control group’s PRS scores, CD-RISC scores, SCI scores, and BEPSI scores before and after the urban garden activity program. For the analysis of the difference in cortisol levels as a physiological indicator of stress, a corresponding paired *t*-test and RM ANOVA were used. All statistical tests used a *p*-value of <0.05 as the significance level.

## 3. Results

### 3.1. Demographic Characteristics

In order to identify the general characteristics of the study participants, frequency analysis was conducted by gender, age, educational background, occupation, and average monthly income. The results of the frequency analysis for this are shown in Table 3. Most of the participants were women (75.6%). A total of 43 participants were in their 50s (4.8%), 24 participants were in their 60s or older (26.7%), and 12 participants were in their 50s or younger (25.5%). For the education level, 52 participants (57.8%) graduated from university, 29 participants (32.2) graduated from graduate school or higher, and nine participants (10.0%) graduated from high school or lower.

Participants’ occupations were housewives (25.6%), students (22.2%), service workers (12.2%), self-employed and CEOs (12.2%), production and technology workers (11.1%), office administrative workers (7.8%), unemployed–others (5.6%), and professional researchers (3.3%). Most of participants’ average monthly income were less than KRW 1 million (25.6%) or between KRW 2 million and KRW 3 million (22.2%).

### 3.2. Perceived Restorativeness

Table 4 shows the results of repeated measurement variance analysis to verify the difference in PRS pre- and post-scores of experimental groups 1 and 2 who participated in the urban garden activity program and the control group. It can be seen that the differences between the four sub-factors of repose, fascination, coherence, and legibility in the restorative environment, the interaction effect between time and group were statistically significant in repose factors (F = 31.009, *p* < 0.001). The scores of experimental group 1 and experimental group 2 increased significantly in the post-test than that of the pre-test.

In addition, the results of repeated measures ANOVA by the group to find out the change in repose factors for each group showed statistically significant differences in experimental group 1 (F = 54.497, *p* < 0.001), experimental group 2 (F = 21.720, *p* < 0.001), and control group (F = 12.231, *p* = 0.002).

The fascination factor’s interaction effect between time and group was statistically significant (F = 11.760, *p* < 0.001). Furthermore, it can be seen that the fascination scores of experimental group 1 and experimental group 2 were increased. In addition, the results of repeated measures ANOVA by the group to find out the change in the fascination factor showed a statistically significant difference in experimental group 1 (F = 47.698, *p* < 0.001) and experimental group 2 (F = 22.077, *p* < 0.001).

The Coherence factor’s interaction effect between time and group was not statistically significant (F = 3.588, *p* = 0.076). Therefore, looking at the results of repeated measures ANOVA for each group to find out the changes in the Coherence factors for each group, statistically significant differences were found in experimental group 1 (F = 26.437, *p* < 0.001) and experimental group 2 (F = 12.878, *p* = 0.001).

In addition, the results of repeated measures ANOVA by the group to find out the changes in the legibility factors showed statistically significant differences in experimental group 1 (F = 49.685, *p* < 0.001) and experimental group 2 (F = 16.761, *p* < 0.001).

### 3.3. Resilienece

Table 5 shows the results of RM ANOVA to verify the difference in CD-RISC pre- and post-score for experimental groups 1 and 2 who participated in the urban garden activity program and the control group. The Hardiness factor’s interaction effect between time and group was statistically significant (F = 11.539, *p* < 0.001). It can be seen that the Hardiness factor scores of experimental group 1 and experimental group 2 were increased. In addition, the results of repeated measures ANOVA by the group to find out the change in the Hardiness factor showed a statistically significant increase in experimental group 1 (F = 51.958, *p* < 0.001) and experimental group 2 (F = 14.466, *p* = 0.001).

In the Persistence factor, the interaction effect between time and group was statistically significant (F = 10.706, *p* < 0.001). It can be seen that the persistence factor scores of experimental group 1 and experimental group 2 were increased. In addition, the results of RM ANOVA by group to find out the change in the persistence factor increased statistically significantly in experimental group 1 (F = 41.321, *p* < 0.001) and experimental group 2 (F = 16.080, *p* < 0.001).

In the optimistic factor, the interaction effect between time and group was statistically significant (F = 7.853, *p* = 0.001). It can be seen that the optimistic factors of experimental group 1 and experimental group 2 were increased. In addition, the results of RM ANOVA by group to find out the change in the optimistic factor was increased statistically significant in experimental group 1 (F = 15.149, *p* = 0.001) and experimental group 2 (F = 25.495, *p* < 0.001).

In the control group, factor’s interaction effect between time and group was not statistically significant (F = 2.431, *p* = 0.094). Therefore, looking at the results of RM ANOVA for each group to find out the change in support factors for each group, experimental group 1 (F = 6.021, *p* = 0.020) and experimental group 2 (F = 6.167, *p* = 0.019) were increased statistically significantly.

In the Spirit factor, the interaction effect between time and group was not statistically significant (F = 1.982, *p* = 0.144). Therefore, the results of RM ANOVA by the group to determine the change in the spirituality factor for each group were significantly increased in experimental Group 1 (F = 12.941, *p* = 0.001) and experimental Group 2 (F = 1.478, *p* = 0.23).

### 3.4. Sense of Community

Table 6 shows the results of RM ANOVA to verify the difference in Sense of Community Index (SCI) pre- and post-score of experimental groups 1 and 2 who participated in the urban garden activity program and the control group. The interaction effect between time and group was statistically significant (F = 13.054, *p* < 0.001). It can be seen that the factors Integration and Fulfillment of needs in experimental group 1 and experimental group 2 were increased. In addition, the results of RM ANOVA for each group to find out the change in the Integration and Fulfillment of needs were increased significantly in experimental group 1 (F = 36.001, *p* < 0.001) and experimental group 2 (F = 26.868, *p* < 0.001).

The interaction effect between time and group was statistically significant in the factor of membership (F = 11.034, *p* < 0.001). It can be seen that the scores of the factors of membership in experimental group 1 and experimental group 2 were increased. In addition, the result of RM ANOVA by the group to find out the change in the factors of membership is experimental group 1 (F = 65.384, *p* < 0.001), experimental group 2 (F = 26.025, *p* < 0.001) showed a statistically significant increase in post-test than pre-test.

The interaction effect between time and group was statistically significant in the factor of influence (F = 3.777, *p* = 0.027). It also can be seen that the factor of influence between experimental group 1 and experimental group 2 was increased. In addition, the results of RM ANOVA by group to find out the change in the factors of Influence showed that experimental group 1 (F = 8.939, *p* = 0.006) and experimental group 2 (F = 26.868, *p* < 0.001) increased statistically significantly in the post-test compared to the pre-test.

In the Shared Emotional Connection factor, the interaction effect between time and group was statistically significant (F = 8.273, *p* < 0.001). It can be seen that the factor scores of Shared Emotional Connection between experimental group 1 and experimental group 2 were increased. In addition, the results of RM ANOVA by the group to find out the change in the factors of Shared Emotional Connection increased statistically significantly in the post-test compared to the pre-test in experimental group 1 (F = 54.696, *p* < 0.001) and experimental group 2 (F = 16.650, *p* < 0.001).

### 3.5. Stress

Table 7 presents the results of stress reduction from gardening activities for each group. The interaction effect between time and group was statistically significant (F = 4.600, *p* = 0.013). It can be seen that there were reductions in stress in experimental group 1 and experimental group 2. In addition, the results of RM ANOVA by the group to find out the changes in stress were significantly reduced in experimental group 1 (F = 41.043, *p* < 0.001) and experimental group 2 (F = 13.476, *p* = 0.001).

### 3.6. Cortisol

#### 3.6.1. Verification of the Difference between Pre- and Post-Cortisol in the Group (Repeated Measurement Variance Analysis)

Considering the circadian rhythm of salivary cortisol, researchers collected a total of four measurements, one pre-test measurement and one post-test measurement in two-hour intervals in the intermediate session. Repeated measures ANOVA was conducted to verify the difference between cortisol according to experimental group 1 and experimental group 2 participating in urban garden activities and the control group.

In order to reduce the variables of the study and measure the effect more accurately, the pre-second and post-third differences measured at the beginning and end of the mid-session were analyzed once more by dividing the analysis into two sessions. As shown in Table 8, the interaction effect between time and group before and after was not statistically significant (F = 0.342, *p* = 0.711). Therefore, looking at the results of verifying the difference in cortisol by performing RM ANOVA again to find out the change in cortisol by group, there was a statistically significant difference in experimental group 1 (F = 13.506, *p* = 0.001). As shown in Table 9, the interaction effects between the group and the time before and after measures in the mid-session were not statistically significant (F = 1.248, *p* = 0.292). Therefore, the results of verifying the difference in cortisol by performing RM ANOVA again to find out the change in cortisol by group, there was a statistically significant difference in experimental group 1 (F = 5.255, *p* = 0.030). As a result of the two analyses, there was a statistically significant decrease only in experimental group 1 and a cortisol decrease in both experimental group 2 and the control group, but no significant difference was found.

#### 3.6.2. Verification of the Difference between Pre- and Post-Cortisol in Homogeneous Groups

As a result of repeated cortisol measurements, the interaction effect between time and group was not statistically significant, so the results of a paired *t*-test are shown in Table 10 to observe if there was a difference in the mean cortisol levels. In the experimental group 1, the cortisol concentration decreased statistically significantly post-test than pretest (*t* = 4.457, *p* < 0.001). In the interim measurement of the session, experimental group 1 (*t* = 2.651, *p* = 0.013) showed a significant difference (Table 11).

## 4. Discussion

This study aimed to investigate the effect of urban garden activities on participants’ psychological and physiological benefits. This study showed that urban garden activities significantly increase participants’ perceived restorativeness. The perceived restorativeness after participation increased statistically significantly in the urban garden activity participating group compared to before participating in the program. However, there was no significant difference for the control group. The results of this study are consistent with those shown by Tyrväinen et al. [33] that short-term visits to urban natural areas have a positive effect on stress relief and restorative environment perception compared to the architectural environment. Kang and Suh [34] also reported that visitors to Suncheon Bay National Garden experience stress relief and restoration naturally through involuntary attention.

This study also showed that urban garden activities significantly increase participants’ resilience. Participants in urban garden activities significantly increased their resilience, but the control group showed no significant change. These results are consistent with the results of Lee et al. [18] who showed that healing agricultural activities for young adults relieve stress and improve resilience and happiness, and An [35] who revealed that physical activities such as outdoor walking for adults have a significant effect on resilience and psychological happiness.

Moreover, this study demonstrated that urban garden activities significantly increase participants’ sense of community. Participants in urban garden activities showed a significantly increased sense of community, but the control group showed no significant change. These results are consistent with the results of Park and Lee [36]. They reported that participants in the urban garden program showed a significant increase in sense of community. Jeong and Jang [37] also reported positive effects of urban garden activities and argued the urban garden as an important place to develop users’ sense of community.

This study showed that urban garden activities significantly decrease participants’ stress levels. Participants in urban garden activities showed a statistically significant decrease in stress after participation compared to before participation in the program. The control group had a decrease in stress; however, the difference was not statistically significant. These results are consistent with Stigsdotter and Grahn’s [38]. They reported people access to gardens have positive effect on stress. Park et al. [39] also demonstrated that healing programs performed in gardens provide stress relief effects in the elderly.

This study showed that there was a significant reduction in cortisol levels after participants engaged in the urban garden activities in experimental group 1. However, for the control group and experimental group 2 there were no statistically differences in cortisol level. These results are consistent with Jang et al. [40] who showed that stress was relieved, and cortisol levels were lowered in adult gardening plant cultivation activities. Therefore, urban garden activities can contribute to the community’s revitalization and have positive effects, such as strengthening participants’ attentional resilience, resilience, and stress relief.

As Lee [41] found that cortisol in subjects who shed tears or expressed upset during cortisol measurement increased, further study is required to verify these findings more specifically. In addition, when measuring cortisol, it is believed that cortisol levels increased while physically struggling as participants skipped water or meals.

This study revealed the effectiveness of the urban garden activity program using gardens for adults. It is meaningful that the garden activity program verified the effectiveness of attention resilience, resilience, increased sense of community, and stress reduction. Therefore, urban garden activities can contribute to community revitalization and positive effects on psychological health, such as increased attention resilience, resilience, and stress relief of urban residents.

In this study, there were some limitations as follows. In order to generalize the research results, first, it is necessary to secure a sufficient number of research participants nationwide for garden activities. We recruited voluntary participants for the experiment via notices posted in community centers and local newspapers. Other methods for volunteer recruitment, such as online notices, would be employed in further studies to secure sampling representation. Second, since there was a difference in the proportion of male and female participants in this study, a study with a similar proportion of subjects should be conducted in subsequent studies. Third, since it has not been verified whether the program’s effect persists even after returning to daily life after the program is terminated, a follow-up study is needed to determine the effect after the program is terminated. Fourth, the intensity of physical activities such as planting and management in the garden was not adjusted according to the size of the garden. In subsequent studies, physical activity should be controlled in gardens of similar sizes.

## 5. Conclusions

This study investigated that urban garden activities have physiological and psychological effects on participants. After 16 sessions of garden activities, the participants of the study showed significant positive changes in their perceived restorativeness, resilience, increased sense of community and decreased stress. Therefore, it is believed that urban garden activities are effective ways to relieve daily stress.

## Figures and Tables

**Table 1:** Research Design for Program Effectiveness Verification.

Group	Pre-Test	Experimental Treatment	Post-Test
Experimental group 1	○	◇, □	◎
Experimental group 2	○	◇	◎
Control group	○		◎

Note: ○, pre-test; ◇, garden activities program 1; □, garden activities program 2; ◎, post-test.

**Table 2:** Urban gardening activities program.

Session	Period	Garden Activity Program I	Garden Activity Program II
1	May Spring	Orientation Garden design Planting spring flower	Proper walking and Walking meditation
2		Placement and Planting of Trees and Herbal plants Vegetable garden planting and management	Breathing meditation
3		Pest management Garden plant sign drawing	Singing Bowl Meditation
4	June Summer	Planting aquatic plants Planting summer plants	Phalaenopsis flower pot making
5		Propagating with Plant Cuttings Harvesting vegetable garden crops	Healthy Meal Garden Farm Party Crop Sharing and Donation
6	July Summer	Flower planting and garden management	Making Herb Scent Bags
7		Understanding and management of medicinal plants	Making Aroma Oil Fragrance Necklace
8	August Summer	Flower bed creation and herb plant management	Aroma massage
9		Planting and arrangement of autumn herbaceous plants Create a vegetable garden	Object meditation
10	September Fall	Garden care and sign drawing	Garden Plant Miniature Drawing 1
11		Autumn herbaceous planting, garden management	Garden Plant Miniature Drawing 2
12	Octorber Fall	Autumn herbaceous planting garden management	Flower tea therapy
13		Pruning and tree care	Making natural dyed scarves Using garden plants
14	November Fall	Autumn bulbous planting	Making rice cakes with local rice
15		Garden Plant Overwintering care Crop harvest	Making a mini garden with houseplants, Crop Sharing and Donation
16		Garden Plant Overwintering care Surveys and Cortisol Measurements	farm party

**Table 3:** General characteristics of participants.

Variable	Category	Frequency	Percent (%)
Gender	Male	22	24.4
	Female	68	75.6
Age	Under 49	12	25.5
	50–59	43	47.8
	Over 60	24	26.7
Education	Less than high school	9	10.0
	Graduated from college	52	57.8
	More than graduate university	29	32.2
Occupation	Office worker	7	7.8
	Production/technician	10	11.1
	Profession/researcher	3	3.3
	Service job	11	12.2
	Self-employed-CEO	11	12.2
	Student	20	22.2
	Housewife	23	25.6
	Unemployed, etc.	5	5.6
Monthly income (won)	Less than 1 million	23	25.6
	1 million ~ less than 2 million (KRW)	16	17.8
	2 million ~ less than 3 million (KRW)	20	22.2
	3 million ~ less than 4 million (KRW)	13	14.4
	4 million ~ less than 5 million (KRW)	6	6.7
	More than 5 million (KRW)	12	13.3
Total		90	100.0

**Table 4:** Change in Perceived Restorativeness scale by variables.

Variables	Group	Pre (Score)		Post (Score)		Time	Group × Time
		M	SD	M	SD		
Repose	Experimental group 1	4.80	1.00	5.71	0.78	F = 54.497, *p* < 0.001	F = 31.009, *p* < 0.001 *η^2^ₚ* = 0.416
	Experimental group 2	5.17	0.91	5.79	0.61	F = 21.720, *p* < 0.001	
	Control group	5.19	0.76	4.64	0.80	F = 12.231, *p =* 0.002	
Fascination	Experimental group 1	4.90	0.98	5.75	0.79	F = 47.698, *p* < 0.001	F = 11.760 *p* < 0.001 *η^2^ₚ* = 0.213
	Experimental group 2	5.24	0.86	5.85	0.69	F = 22.077, *p* < 0.001	
	Control group	5.14	0.98	4.91	0.81	F = 1.048, *p =* 0.314	
Coherence	Experimental group 1	5.50	1.03	6.18	0.62	F = 26.437, *p* < 0.001	F = 3.588 *p =* 0.076
	Experimental group 2	5.48	1.06	6.01	0.73	F = 12.878, *p =* 0.001	
	Control group	5.50	1.27	5.50	0.94	F = 1.000, *p* = 1.00	
Legibility	Experimental group 1	5.00	0.91	5.83	0.76	F = 49.685, *p* < 0.001	F = 8.670 *p* < 0.001 *η^2^ₚ* = 0.166
	Experimental group 2	5.08	1.16	5.66	0.88	F = 16.761, *p* < 0.001	
	Control group	5.00	1.20	4.83	1.07	F = 0.467, *p =* 0.500	

**Table 5:** Changes in the Connor–Davidson Resilience Scale by variables.

Variables	Group	Pre (Score)		Post (Score)		Time	Group × Time
		M	SD	M	SD		
Hardiness	Experimental group 1	2.62	0.37	3.00	0.43	F = 51.958, *p* < 0.001	F = 11.539 *p* < 0.001 *η^2^ₚ* = 0.210
	Experimental group 2	2.60	0.55	2.88	0.56	F = 14.466, *p =* 0.001	
	Control group	2.68	0.42	2.66	0.36	F = 0.192, *p =* 0.664	
Persistence	Experimental group 1	2.84	0.36	3.25	0.41	F = 41.321, *p* < 0.001	F = 10.706 *p* < 0.001 *η^2^ₚ* = 0.197
	Experimental group 2	2.91	0.55	3.15	0.45	F = 16.080, *p* < 0.001	
	Control group	3.03	0.51	3.05	0.44	F = 0.101, *p =* 0.753	
Optimism	Experimental group 1	2.93	0.43	3.22	0.51	F = 15.149, *p* = 0.001	F = 7.853 *p =* 0.001 *η^2^ₚ* = 0.153
	Experimental group 2	2.69	0.56	3.03	0.44	F = 25.495, *p* < 0.001	
	Control group	2.99	0.54	2.96	0.45	F = 0.205, *p =* 0.654	
Control	Experimental group 1	2.88	0.45	3.12	0.55	F = 6.021, *p* = 0.020	F = 2.431 *p* = 0.094
	Experimental group 2	2.90	0.61	3.08	0.49	F = 6.167, *p* = 0.019	
	Control group	3.03	0.60	3.00	0.60	F = 0.108, *p* = 0.745	
Spirit	Experimental group 1	2.37	0.78	2.68	0.68	F = 49.685, *p* < 0.001	F = 8.670 *p* < 0.001
	Experimental group 2	2.50	0.69	2.63	0.72	F = 16.761, *p* < 0.001	
	Control group	2.48	0.52	2.55	0.63	F = 0.467, *p =* 0.500	

**Table 6:** Changes in Sense of Community Index scale by variables.

Variables	Group	Pre (Score)		Post (Score)		Time	Group × Time
		M	SD	M	SD		
Integration and Fulfillment of needs	Experimental group 1	3.61	0.55	4.13	0.49	F = 36.001, *p* < 0.001	F = 13.054 *p* < 0.001 *η^2^ₚ* = 0.231
	Experimental group 2	3.68	0.47	4.09	0.44	F = 26.868, *p* < 0.001	
	Control group	3.88	0.50	3.79	0.65	F = 0.750, *p =* 0.394	
Membership	Experimental group 1	2.78	0.48	3.90	0.56	F = 65.384, *p* < 0.001	F = 11.034 *p* < 0.001 *η^2^ₚ* = 0.202
	Experimental group 2	3.22	0.54	3.78	0.60	F = 26.025, *p* < 0.001	
	Control group	3.26	0.44	3.51	0.59	F = 0.750, *p =* 0.091	
Influence	Experimental group 1	2.78	0.47	3.19	0.55	F = 8.939, *p =* 0.006	F = 3.777 *p* = 0.027 *η^2^ₚ* = 0.080
	Experimental group 2	2.76	0.62	3.02	0.47	F = 26.868, *p* < 0.001	
	Control group	2.87	0.46	2.91	0.45	F = 0.219, *p =* 0.643	
Shared Emotional Connection	Experimental group 1	3.82	0.43	4.33	0.38	F = 54.696, *p* < 0.001	F = 8.723 *p* < 0.001 *η^2^ₚ* = 0.167
	Experimental group 2	3.88	0.60	4.21	0.45	F = 16.650, *p* < 0.001	
	Control group	3.88	0.63	3.88	0.61	F = 1.000, *p =* 0.998	

**Table 7:** Changes in the Brief Encounter Psychosocial Instrument by variable.

Variables	Group	Pre (Score)		Post (Score)		Time	Group × Time
		M	SD	M	SD		
Stress	Experimental group 1	2.46	0.75	1.89	0.53	F = 41.043, *p* < 0.001	F = 4.600 *p* = 0.013 *η^2^ₚ* = 0.096
	Experimental group 2	2.37	0.70	1.87	0.46	F = 13.476, *p =* 0.001	
	Control group	2.25	0.72	2.16	0.56	F = 0.986, *p =* 0.520	

**Table 8:** Results of repeated measures ANOVA of the difference between cortisol before and after in the groups.

Variables	Group	Pre (Score)		Post (Score)		Time	Group × Time
		M	SD	M	SD		
Cortisol	Experimental group 1	0.170	0.076	0.105	0.055	F = 13.506, *p* = 0.001	F = 0.342 *p* = 0.711
	Experimental group 2	0.182	0.187	0.132	0.100	F = 1.800, *p* = 0.190	
	Control group	0.147	0.084	0.112	0.069	F = 4.166, *p* = 0.051	

**Table 9:** Results of repeated measures ANOVA of the difference between mid-session in the groups.

Variables	Group	Pre (Score)		Post (Score)		Time	Group × Time
		M	SD	M	SD		
Cortisol	Experimental group 1	0.127	0.084	0.084	0.052	F = 5.255, *p* = 0.030	F = 0.1.248 *p* = 0.292
	Experimental group 2	0.141	0.089	0.139	0.071	F = 0.110, *p* = 0.919	
	Control group	0.166	0.090	0.140	0.088	F = 2.027, *p =* 0.166	

**Table 10:** Differences in cortisol before and after urban gardening program.

Variables	Group	Pre		Post		*t*	*p*
		M	SD	M	SD		
Cortisol	Experimental group 1	0.180	0.077	0.100	0.054	4.457	0.000
	Experimental group 2	0.182	0.186	0.132	0.099	1.342	0.190
	Control group	0.146	0.083	0.111	0.068	2.041	0.051

**Table 11:** Differences in cortisol before and after mid-session in urban gardening program.

Variables	Group	Pre		Post		*t*	*p*
		M	SD	M	SD		
Cortisol	Experimental group 1	0.131	0.081	0.083	0.051	4.457	0.000
	Experimental group 2	0.140	0.089	0.138	0.071	1.342	0.190
	Control group	0.165	0.090	0.140	0.088	2.041	0.051

## Data Availability

Not applicable.
